# Cutaneous Metastasis in Breast Cancer: A Case Series

**DOI:** 10.7759/cureus.40109

**Published:** 2023-06-08

**Authors:** Neelesh Shrivastava, A Balasubramanian

**Affiliations:** 1 Surgical Oncology, Jawaharlal Institute of Postgraduate Medical Education and Research, Pondicherry, IND

**Keywords:** neoadjuvant chemotherapy (nact), disease-free interval (dfi), progesterone receptor (pr), human epidermal growth factor receptor 2 (her2), estrogen receptor (er), immunohistochemistry (ihc), outpatient department (opd), carcinoma erysipeloides

## Abstract

The most frequent reason for cutaneous metastases is breast cancer in females. Breast cancer patients can present with cutaneous manifestations of breast disease at the time of their initial diagnosis; however, cutaneous metastases more often present well after the initial diagnosis and treatment of the breast disease. We described three cases of carcinoma of breast metastasis to the skin of the breast and the chest wall, each with a unique dermatological presentation. A 52-year-old woman presented with a cutaneous erythematous papule for the past month. She underwent a modified radical mastectomy one year before. On presentation, she was diagnosed to have erythematous papule near the operative scar and surrounding chest wall and referred to the dermatology outdoor department, where a skin biopsy was done, which confirmed erysipeloides carcinoma. The second case includes a 38-year-old premenopausal lady who was diagnosed with carcinoma of the right breast with a locally advanced stage. She was treated with neoadjuvant chemotherapy (NACT) followed by modified radical mastectomy and subsequently presented with biopsy-proven multiple skin nodules on the chest wall at the same side. She was discussed in a multidisciplinary tumor board and planned for palliative chemotherapy followed by hormonal therapy. In the third case, a 42-year-old perimenopausal woman diagnosed with locally advanced left breast carcinoma presented in the surgical oncology outdoor patient department (OPD) with multiple skin erythema over the left breast. Biopsy was done from the skin erythema site showing metastasis to the skin. She was discussed in a multidisciplinary tumor board and planned for systemic chemotherapy followed by assessment for surgery. Skin erythema and erythematous papules are rare manifestations of cutaneous metastasis in patients with carcinoma of the breast; typically, patients present with a chest wall nodule. Careful examination and early detection of these uncommon skin lesions can lower morbidity and slow the progression of diseases in these patients.

## Introduction

Breast cancer is the most common cancer associated with cutaneous metastasis in females [1]. The presence of cutaneous metastasis is an indicator of the systemic spread of primary disease and is associated with poor prognosis [2]. Breast cancer is a heterogeneous disease, and due to molecular differences, histologically similar cancers can have different responses to therapy and different prognosis [3]. The most common site for skin metastasis includes the anterior chest wall; except this site, other sites of skin metastasis include the contralateral breast, scar sites, arms, and the head and neck regions [3]. Skin metastasis of solid tumors accounts for 2% of all skin tumors [3]. The incidence of breast carcinoma cutaneous metastases in patients with breast carcinoma is 23.9% [4]. The spectrum of presentation of cutaneous metastasis in breast cancer varies in frequency from the common papulonodular variant to much rarer presentations of dermatitis-like metastases [5]. With an emphasis on the various clinical presentations of the disease's dermatological patterns, we present three cases of breast cancer with metastasis to the skin.

## Case presentation

Case first (triple-negative subtype)

A 52-year-old postmenopausal woman presented with erythema and erythematous papules on the left side of the chest wall for the last month. She was diagnosed with a case of carcinoma of the left breast two years before, her clinical finding was a 6x5 cm lump in the left upper outer quadrant of the breast and a 1x1 cm palpable mobile axillary lymph node in the left axilla, and her clinical stage was T3N1M0. She underwent a core needle biopsy of the breast lump which shows infiltrating ductal carcinoma with immunohistochemistry showing estrogen receptor-negative, progesterone receptor-negative, and her 2 receptors negative subtype. In view of locally advanced disease, she was discussed in the multidisciplinary tumor board and planned for neoadjuvant chemotherapy followed by surgery. Before starting neoadjuvant chemotherapy, her metastatic workup was done which shows no distant metastasis (via positron emission tomography-computed tomography scan). She had a modified radical mastectomy, and postoperative histology revealed infiltrating ductal carcinoma, grade 2, score 6, all margins negative with all lymph nodes negative. She had finished post-operative radiation therapy and was being monitored.

**Figure 1:**
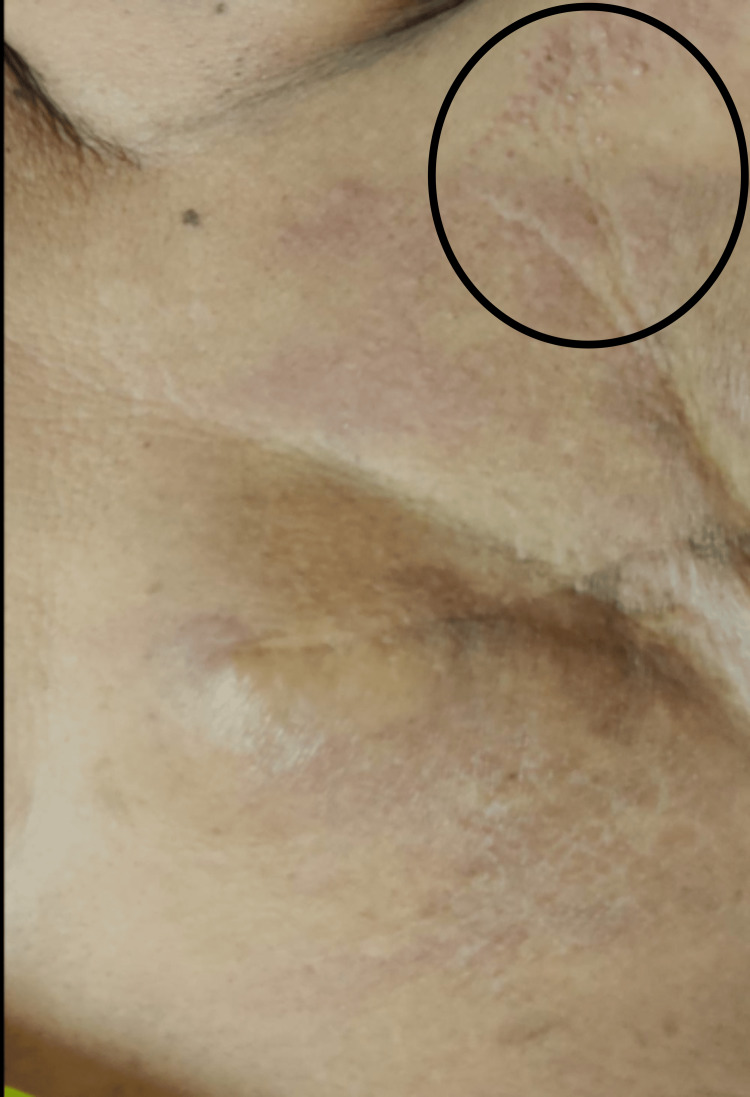
Erythema over the left chest wall with multiple papules over the superior aspect

She self-referred to the dermatology outpatient department after a disease-free interval of one year with symptoms of erythema and erythematous papules over the left chest wall that have been progressively becoming worse over the last month and spreading across the chest wall. It had no discomfort or pruritus attached to it. Physical examination of the patient reveals widespread erythema and erythematous papules throughout the left chest wall, numerous tiny papules that are firm to the touch, and no apparent ulcers at the afflicted region. The lesion was limited to the left chest wall alone, and no nodules were above the scar site (Figure 1).

A skin biopsy revealed carcinoma erysipeloides. She was discussed in the multidisciplinary tumor board and planned for palliative chemotherapy (IV paclitaxel 60 mg/m^2^ every week). After starting on palliative chemotherapy, the patient responded well with three cycles of chemotherapy and her erythema resolved; she is now on palliative chemotherapy and doing well.

Case second (triple-positive subtype)

**Figure 2:**
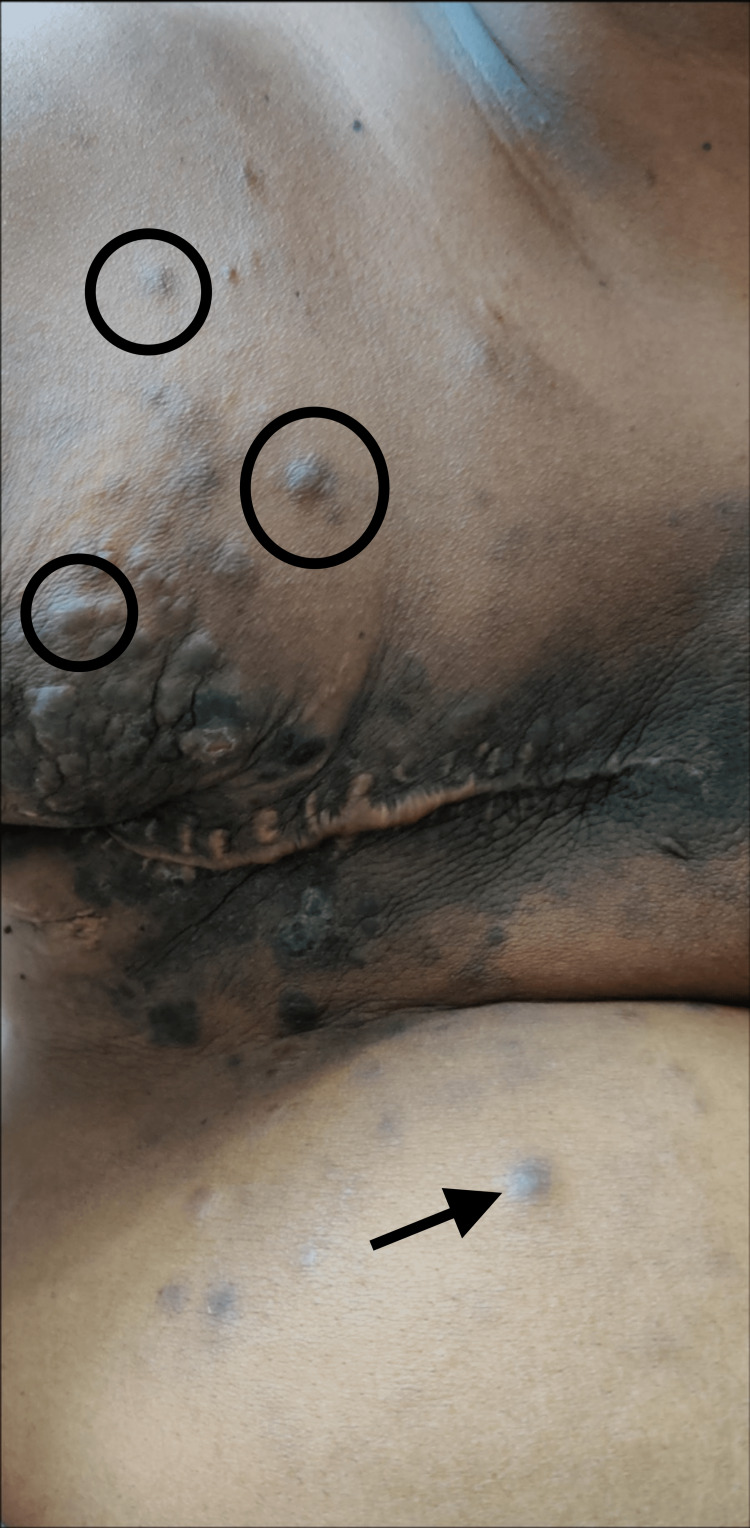
Multiple subcutaneous nodules over the chest wall and abdominal wall

A 38-year-old premenopausal woman presented with a lump in the right breast, and her clinical finding was a 4x4 cm lump with 1x1 cm skin ulceration in the right upper and central quadrant of the breast and a 2x1 cm mobile lymph node in the right axilla. Incision biopsy from the ulcer edge confirmed infiltrating ductal carcinoma, grade 3 with estrogen receptor-positive, progesterone receptor-positive, and her 2 receptor-positive hormonal receptor status. Her clinical stage was cT4bN1M0. She was discussed in the multidisciplinary tumor board and planned for neoadjuvant chemotherapy followed by surgery. Before starting neoadjuvant chemotherapy, her metastatic workup was done which shows no distant metastasis (via positron emission tomography-computed tomography scan). She had four cycles of docetaxel and three cycles of 5-fluorouracil+epirubicin+cyclophosphamide. She underwent a right-sided modified radical mastectomy. She was diagnosed to have bone metastases during the follow-up visits in the outpatient department (metastasis on the L2 vertebra). She was started on tamoxifen (20 mg every day), zoledronic acid (4 mg once a month ), and Herceptin (8 mg/kg stat dose followed by 6 mg/kg every 21 days). She also underwent palliative radiation therapy (RT) 8 Gy for her lumbar spine and hemi-pelvis. She was presented with multiple tiny nodules over the right chest wall for the last three months (Figure 2).

An excision biopsy of the nodule confirms the cutaneous metastasis from breast cancer. In view of the metastatic disease, she was again discussed in the multidisciplinary tumor board and planned for palliative chemotherapy with hormonal therapy (tamoxifen 20 mg once a day) with trastuzumab (6 mg/kg every 21 days). She initially responded to the treatment but ultimately progressed and died within two months of palliative treatment.

Case third (HER2-enrich subtype)

**Figure 3:**
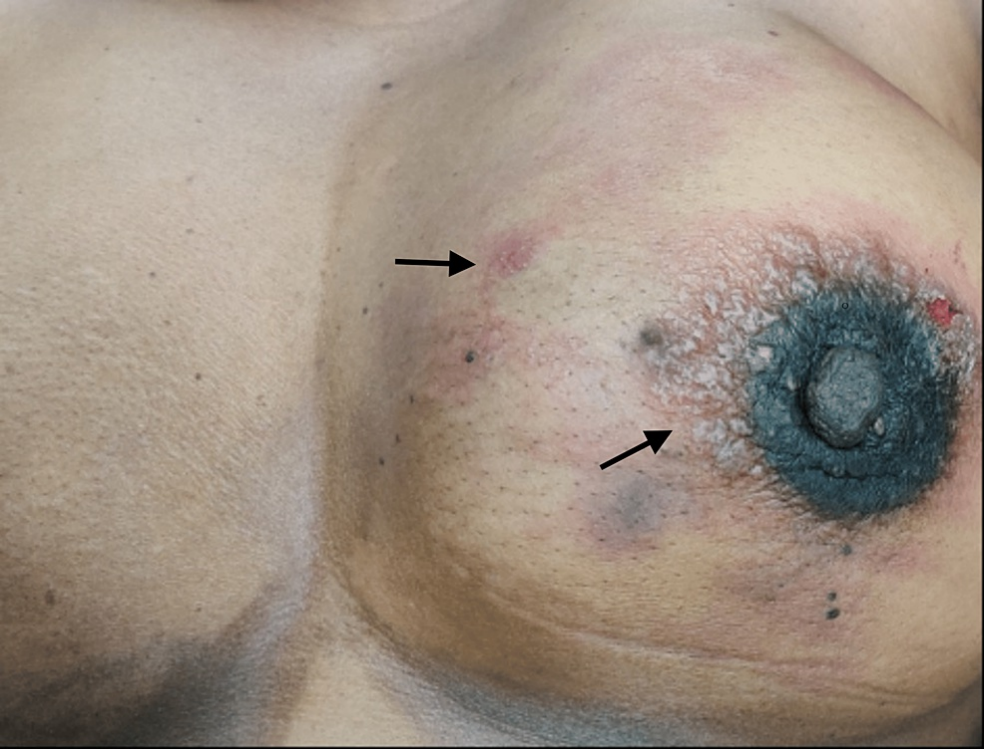
Erythema over the left breast

A 42-year-old perimenopausal woman presented with a 6x6 cm lump with peau d'orange involving all four quadrants of the left breast with matted fixed lymph nodes in the left axilla. She underwent a core needle biopsy of the left breast lump which was reported as infiltrating ductal carcinoma, grade 2 and immunohistochemistry reported estrogen receptor-negative, progesterone receptor-negative, and her 2 receptor-positive subtypes. Her clinical stage was cT4bN2aM0. She was discussed in the multidisciplinary tumor board and planned for neoadjuvant chemotherapy and targeted therapy (trastuzumab 4 mg/kg) followed by surgery. Before starting neoadjuvant chemotherapy, her metastatic workup was done which shows no distant metastasis (via positron emission tomography-computed tomography scan). After completion of chemotherapy, she defaulted on the treatment and presented after three months with the complaint of a lump in her left breast and erythema all over her breast and chest. Her repeat metastatic workup was negative, and a skin biopsy revealed metastatic carcinoma. She was discussed in a multidisciplinary tumor board and planned for systemic chemotherapy and targeted therapy (trastuzumab 6mg/kg every 14 days) followed by an assessment of surgery (Figure 3). After completion of systemic chemotherapy and targeted chemotherapy, the patient responded well and underwent a modified radical mastectomy on the left side. She is now on targeted therapy and follow-up.

## Discussion

Breast cancer and lung cancer are the most common primary organ which spread to the skin [2]. Cutaneous metastasis in solid tumors is generally present in the later stage, but in breast cancer, it depends on the molecular subtype [3]. Patients with triple-negative subtype and HER2-enrich variety can even present in an early stage of the disease [3]. Patients with hormone receptor-positive subtypes are generally present late in the course of the disease and treated with hormonal therapy. If cutaneous metastasis does not respond to hormonal treatment, chemotherapy can be considered in these subtypes [3]. The total prevalence of cutaneous cancer is 5.3% [4]. Breast cancer cutaneous metastases occur in 23.9% of individuals with breast carcinoma [5].

Most metastases originate in the skin and appear at the overlaying area or proximal to the initial tumor, with lymphatic dissemination of tumor cells being the most common cause [5]. The clinical symptoms of cutaneous metastases are diverse. Skin nodules are the most common presentation; other presentations include inflammatory (carcinoma erysipeloides) breast cancer and Paget's disease of the nipple. Dermatitis-like metastasis is a very rare presentation of cutaneous metastasis in breast cancer [5].

In our case series, the first case reported cutaneous metastasis after one year of complete treatment. Cho et al. in their study reported two cases of triple-negative cancer which developed skin metastasis early within four months of completed treatment [3]. Papules are not a common presenting spectrum in cutaneous metastasis due to breast cancer. Miguel et al. in their case report mention the erythematous papules as an initial cutaneous metastasis presentation in breast cancer patients [1]. Supporting the above studies our first case presented with erythematous papules as an initial symptom of skin involvement by the primary tumor.

A biopsy of the skin nodule, erythematous lesion, or papule will aid in the diagnosis, and microscopic features will indicate the likely tissue of origin. The histological characteristics of metastasis are typically the same as those of the parent tumor [6]. All three reported cases in the series are having biopsy-proven metastasis to the skin.

Two cases that are discussed in our case series were having Her2 receptor positivity: one of the patients died after three months of diagnosis of skin metastasis, and another one is still alive and responded better with chemotherapy. Cho et al., in their study, show that Her2-enriched breast cancer has a better response with trastuzumab and reappears early if treatment is stopped [3]. In our second case even after starting the trastuzumab, the second case continues to progress; however, the third case responds well to the same therapy, which shows the diversity in the treatment response for the same receptor status in different patients. The prognosis of the patient with cutaneous metastasis is determined by the primary tumor histopathological features and molecular subtype. However, in the majority of cases, who present with an advanced stage, the anticipated survival is usually less than one year at the time of diagnosis of skin metastasis [5].

In the majority of cases when skin metastasis has occurred, the original malignancy has progressed and is therefore untreatable surgically. Depending on the histological features of the initial tumor, different therapies like external beam radiation, chemotherapy, and hormone therapy are employed. Local radiation treatment is a valuable strategy for symptomatic management in some patients with a large breast tumor with ulceration [3]. Palliative treatment is provided to individuals with lesions in which primary tumors are unresectable. This includes keeping the lesion dry and clean and debriding it if it bleeds. To avoid spreading infection, use a hydrocolloid dressing [6].

## Conclusions

A thorough examination and early identification can reduce morbidity and decrease the progression of illnesses in patients with breast cancer. The development of skin erythema or nodules may be a symptom of cutaneous metastases. In order to properly diagnose and treat the loco-regional as well as a systemic disease, clinicians should evaluate patients with a high index of suspicion carefully and subject them to a skin biopsy, especially if they have a history of malignancy and have had a skin lesion for a long time. When treating a patient with such a rare disease, coordination between the clinical specialties is essential for improved patient care.
